# Plasma Proteomic Analysis Distinguishes Severity Outcomes of Human Ebola Virus Disease

**DOI:** 10.1128/mbio.00567-22

**Published:** 2022-04-21

**Authors:** Arthur Viodé, Kinga K. Smolen, Benoit Fatou, Zainab Wurie, Patrick Van Zalm, Mandy Kader Konde, Balla Moussa Keita, Richard Amento Ablam, Eleanor N. Fish, Hanno Steen

**Affiliations:** a Department of Pathology, Boston Children’s Hospital, Boston, Massachusetts, USA; b Harvard Medical School, Boston, Massachusetts, USA; c Precision Vaccines Program, Boston Children’s Hospital, Boston, Massachusetts, USA; d Division of Infectious Diseases, Boston Children’s Hospital, Boston, Massachusetts, USA; e Sustainable Health Foundation (FOSAD), Conakry, Guinea; f Center of Excellence for Training on Research and Priority Diseases (CEFORPAG), Conakry, Guinea; g Toronto General Hospital Research Institute, University Health Network, Toronto, Canada; h Department of Immunology, University of Toronto, Toronto, Canada; National Institute of Allergy and Infectious Diseases

**Keywords:** Ebola virus disease, interferon, plasma proteome, mass spectrometry, Ebola virus

## Abstract

Ebola virus (EBV) disease (EVD) is a highly virulent systemic disease characterized by an aggressive systemic inflammatory response and impaired vascular and coagulation systems, often leading to uncontrolled hemorrhaging and death. In this study, the proteomes of 38 sequential plasma samples from 12 confirmed EVD patients were analyzed. Of these 12 cases, 9 patients received treatment with interferon beta 1a (IFN-β-1a), 8 survived EVD, and 4 died; 2 of these 4 fatalities had received IFN-β-1a. Our analytical strategy combined three platforms targeting different plasma subproteomes: a liquid chromatography-mass spectrometry (LC-MS)-based analysis of the classical plasma proteome, a protocol that combines the depletion of abundant plasma proteins and LC-MS to detect less abundant plasma proteins, and an antibody-based cytokine/chemokine multiplex assay. These complementary platforms provided comprehensive data on 1,000 host and viral proteins. Examination of the early plasma proteomes revealed protein signatures that differentiated between fatalities and survivors. Moreover, IFN-β-1a treatment was associated with a distinct protein signature. Next, we examined those proteins whose abundances reflected viral load measurements and the disease course: resolution or progression. Our data identified a prognostic 4-protein biomarker panel (histone H1-5, moesin, kininogen 1, and ribosomal protein L35 [RPL35]) that predicted EVD outcomes more accurately than the onset viral load.

## INTRODUCTION

The 2013–2016 Zaire Ebola virus (EBV) outbreak in West Africa showed its epidemic potential, with 28,646 cases and 11,323 deaths (1, 2). Initial EBV infection presents with nonspecific symptoms such as fever and fatigue, resembling a variety of endemic diseases, including influenza, malaria, shigellosis, or typhoid fever, rendering differential diagnosis and early isolation challenging (3). As the viral infection progresses, Ebola virus disease (EVD) is characterized by more specific symptoms such as gastrointestinal involvement, hemorrhagic manifestations, and, in severe cases, multiorgan dysfunction or death.

At the time of this outbreak, no vaccine or treatment had been approved for EVD. EBV inhibits the host immune response by encoding in its genome proteins that specifically target the host interferon (IFN) response. VP35 inhibits the production of type I IFN (4), VP24 binds to the transcription factor STAT1 (signal transducer and activator of transcription 1) required for IFN-induced gene activation (5), and VP40 induces the dysregulation and apoptosis of immune cells (6). EVD is marked by an exaggerated systemic inflammatory response and impaired vascular and coagulation systems (7). Studies investigating the immune responses in EVD patients identified notable differences between those who survived and those who died (8, 9). Fatal cases showed a diminished capacity to mount an appropriate immune response, resulting in high viremia, increased proinflammatory cytokine production (10–13), profound lymphocyte apoptosis, suppressed B and CD8^+^ T cell responses (14), and reduced antibody titers (9). In contrast, survivors mounted a robust innate immune response to eliminate infection, exhibited lower levels of viremia, controlled cytokine production, increased NK cells, had appropriate B and CD8^+^ T cell responses (14), and had higher levels of neutralizing antibodies (9). A comprehensive and quantitative characterization of the plasma proteome from EVD cases with different outcomes may identify targets for therapeutic intervention. Blood plasma is ideal for such an undertaking as it is protein rich and directly involved in modulating the immune response, ideally suited to study the host response to infection.

In this study, we report an in-depth characterization of the plasma proteome changes in EVD patients throughout their disease via analysis of 38 sequential samples collected from 12 patients: 8 survivors and 4 fatalities. Nine of these EVD patients were treated with IFN-β-1a (15) based on the results of preclinical studies, in which we had identified IFN-β-1a as an effective inhibitor of EBV infection (16).

Our analytical strategy combined three protein-based platforms covering three plasma subproteomes: the classical plasma proteome, a depleted plasma proteome enriched in tissue leakage-derived proteins, and cytokine/chemokine analyses. We used liquid chromatography-mass spectrometry (LC-MS)- and antibody-based assays, respectively. For the depletion of the most abundant plasma proteins, we treated the plasma samples with perchloric acid (17), which has proven its robustness in a large-scale coronavirus disease 2019 (COVID-19)-focused study with thousands of plasma samples (18).

We identified differences in the plasma proteomes in the early host response between fatalities and survivors. Moreover, we were able to stratify patients based on their protein expression profiles associated with viral loads. Our plasma proteome maps allowed us to derive prognostic biomarkers of outcomes (fatalities versus survivors) that exceeded the performance of viral loads. Finally, we evaluated the effect of IFN-β-1a treatment on the plasma proteome and provide evidence for IFN-mediated changes.

## RESULTS

**FIG 1:**
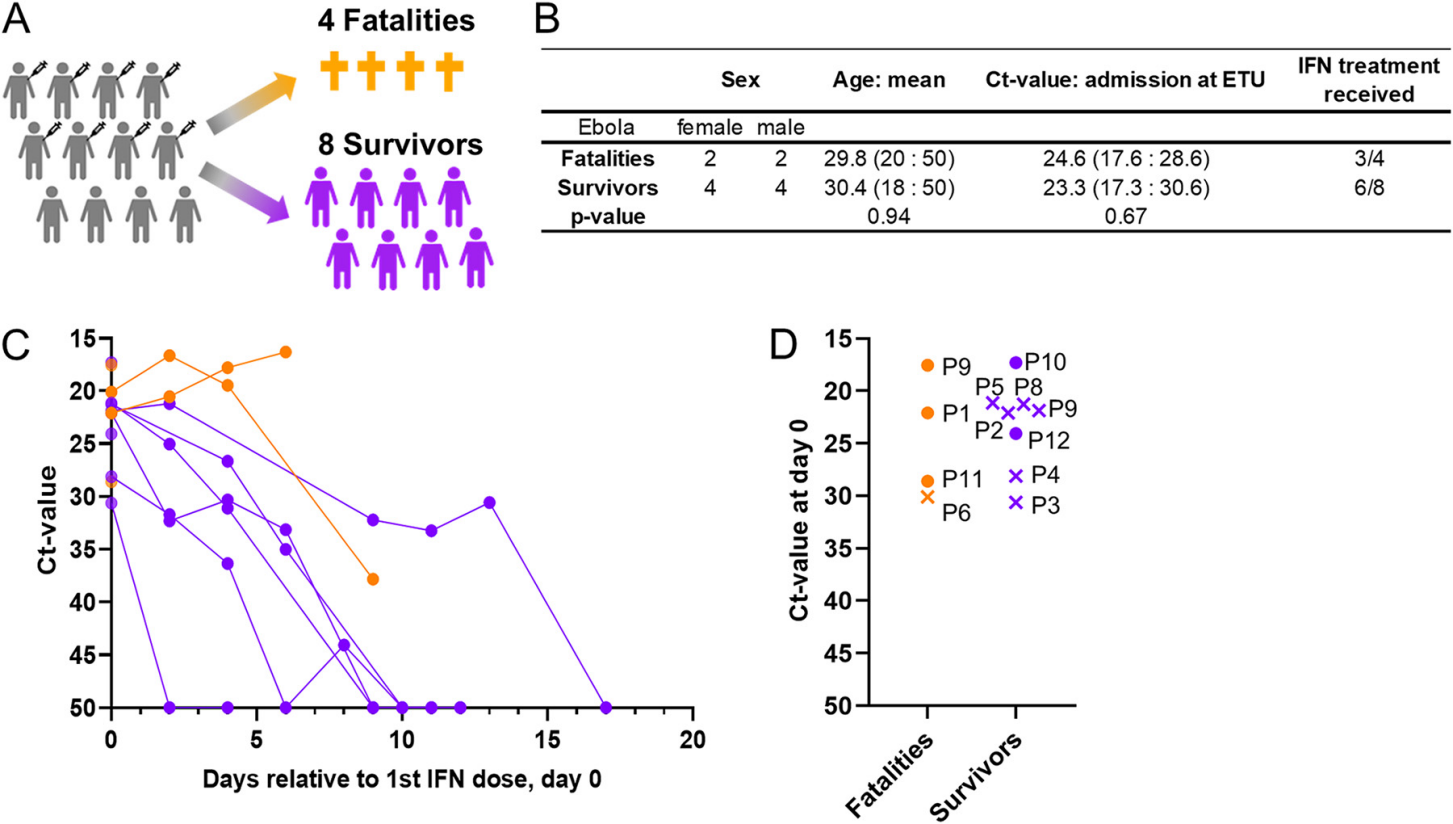
Cohort design and patient demographics. (A) Study cohort outcomes. Twelve patients were included in the study: 8 survivors (purple) and 4 fatalities (orange). (B) Demographic table depicting the characteristics of the study participants. (C) Trajectory of the *C_T_* value, the viral load measured by PCR, for each patient relative to the 1st interferon (IFN) dose (day 0) over the disease course. (D) *C_T_* value at day 0 (1st IFN dose) for survivors (purple) and fatalities (orange). Patients for whom *C_T_* values were obtained but no sample was collected are represented by x’s.

At the time of the 2013–2016 EVD outbreak in Guinea, we undertook a single-arm clinical study to evaluate the safety and therapeutic efficacy of IFN-β-1a treatment. Eligible patients (with confirmed EVD and >18 years of age) received a daily dose of 30 μg IFN-β-1a (6 million international units) by subcutaneous administration (15). Here, we analyzed the plasma proteomes of 38 blood samples from a cohort of 12 EVD patients, 9 of whom were treated with IFN-β-1a (Fig. 1), to better understand the outcome-associated immunophenotypes, develop prognostic biomarkers, and obtain insights into potential drug targets. See Table S1 in the supplemental material for patient characteristics.

10.1128/mbio.00567-22.6 TABLE S1 Characterization of IFN-treated and untreated patients. Download Table S1, XLSX file, 0.01 MB. Copyright © 2022 Viodé et al. 2022 Viodé et al. https://creativecommons.org/licenses/by/4.0/ This content is distributed under the terms of the Creative Commons Attribution 4.0 International license.

Unbiased analysis of the plasma proteome using, e.g., LC-MS-based methods is challenging, as a few proteins account for 90% of the total protein content of plasma, with serum albumin alone accounting for ∼55% of the total protein content (19). Proteomic methods without depletion are unable to detect less abundant plasma proteins, identifying only the most abundant, “classical” plasma proteins (19). To circumvent the bias toward abundant proteins, we depleted the most abundant proteins using perchloric acid precipitation to reveal tissue leakage proteins of lesser abundances (see the workflow in Fig. S1A).

10.1128/mbio.00567-22.2 FIG S1 Proteomic method and analytical workflow of the reproducibility test. (A) Depleted plasma proteomic platform protocol overview. (B, left) Venn diagram comparison of proteins identified by depleted PP and neat PP. (Right) Plasma protein concentrations of identified proteins. Median concentrations are 1.5 μg/mL for neat PP and 0.05 μg/mL for depleted PP. (C) To ensure a reproducible workflow (i.e., precipitation, desalting, digestion, and analysis), a pooled sample was processed five times, and 5 technical replicates were subsequently analyzed. On average, 552 proteins were identified in each replicate, with a total number of 587 proteins identified. Of these 587 proteins, 498 (85%) had no missing values compared across the five replicates, while 37 (6%) of the proteins had one missing value, 29 (5%) had two missing values, 13 (2%) had three missing values, and 10 (2%) were identified in only one sample. The median coefficient of variation (CV) of the LFQ intensity of the proteins was 14.5%, highlighting the robustness of the methods, most likely due to the extreme conditions, which are immune to minor variations. The CV of the LFQ intensity of each protein was calculated across the 5 replicates. Download FIG S1, TIF file, 0.9 MB. Copyright © 2022 Viodé et al. 2022 Viodé et al. https://creativecommons.org/licenses/by/4.0/ This content is distributed under the terms of the Creative Commons Attribution 4.0 International license.

First, we compared our well-established, non-depletion-based/MStern blotting-based protocol for the detection of the classical plasma proteome (20, 21) (classical plasma proteomics [Cl-PP] protocol) to our new high-throughput (HTP) depletion-based plasma proteomics protocol (tissue leakage plasma proteomics [TL-PP] protocol). The Cl-PP protocol identified 371 proteins, with a concentration range from 42 mg/mL to 1.2 ng/mL (22) and a median detected plasma protein concentration of 1.5 μg/mL, consistent with previous studies (20). Compared to the Cl-PP protocol, the total number of proteins identified using the TL-PP protocol nearly doubled, from 371 to 831 (Fig. S1B). Furthermore, the dynamic range of the identified proteins using the TL-PP protocol spans 8 orders of magnitude, from 42 mg/mL down to 0.2 ng/mL, with a median concentration of 0.05 μg/mL (versus 1.5 μg/mL for the Cl-PP protocol). Although the TL-PP protocol led to the quantification of over 800 proteins, including many at significantly lower median concentrations (Fig. S1C), we observed a robust median coefficient of variation (CV) of 14.5%, even after perchloric acid treatment. Approximately 25% (i.e., 201 of 831) of the proteins detected using the TL-PP protocol overlapped the proteins detected using the Cl-PP protocol, demonstrating the complementary nature of both protocols.

### Different immune response signatures between EVD survivors and fatalities.

**FIG 2:**
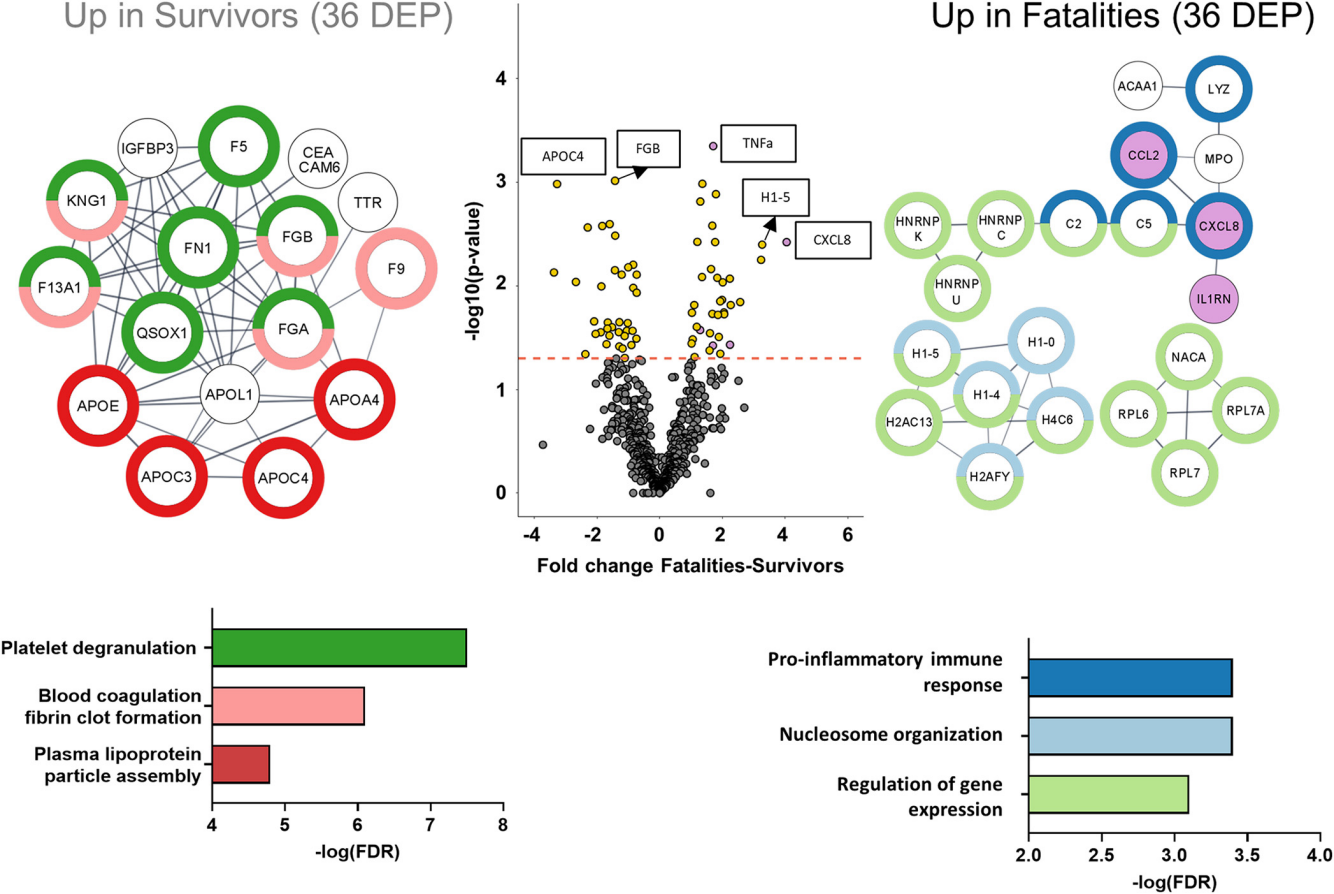
The proteomes of EVD survivors and fatalities are distinguishable early in the disease. Shown is a volcano plot of differentially expressed proteins (DEP) between fatalities and survivors for the first time point collected (survivors, 2 samples at day 0 and 6 samples at day 2 relative to the 1st IFN dose; fatalities, 4 samples at day 0 and 1 sample at day 2). Proteins significantly differentially expressed are represented by a yellow circle, while nonsignificant expression is represented in gray. The dashed red line indicates the *P* value cutoff at 0.05. Gene names of the differentially expressed proteins are represented in the pathway analysis on each side of the volcano plot (left, upregulated in survivors; right, upregulated in fatalities). On the left, the 36 proteins found to be differentially expressed in survivors are associated with platelet degranulation, blood coagulation, fibrin clot formation, and plasma lipoprotein assembly pathways. On the right, the 36 proteins found to be differentially expressed in fatalities were associated with the humoral immune response, nucleosome organization, and regulation of gene expression pathways (*n* = 12) (survivors are in purple; fatalities are in orange).

To characterize the early host response to EBV infection upon admission to the Ebola Treatment Unit (ETU), independent of IFN treatment, we generated a protein matrix based on the three assay platforms. Given the small sample size, we considered the first plasma sample collected per patient (day 0 or 2 relative to the 1st IFN dose) for the early disease outcome signatures. We identified 72 proteins that were significantly differentially expressed (*P* value of <0.05) between fatalities and survivors, with 36 proteins being upregulated in fatalities and 36 proteins being upregulated in survivors (Fig. 2; Table S2).

10.1128/mbio.00567-22.7 TABLE S2 List of proteins associated with EVD survivors and fatalities, which differentiate early in disease progression. Download Table S2, XLSX file, 0.01 MB. Copyright © 2022 Viodé et al. 2022 Viodé et al. https://creativecommons.org/licenses/by/4.0/ This content is distributed under the terms of the Creative Commons Attribution 4.0 International license.

Proteins upregulated in the fatal-outcome group are primarily linker and core histones (H1-4, H1-5, H4-16, H2AC11, and H1-0), ribosomal proteins (ribosomal protein L7 [RPL7], RPL7A, and RPL6) and complement pathway proteins (complement component 5 [C5], C2, complement factor H-related protein 1 [CFHR5], and lysozyme [LYZ]) (Table S2). Using the STRING interaction network for biological pathway analysis (23), we show the enrichment of biological processes involved in the regulation of gene expression (false discovery rate [FDR] of 8.0E−3), nucleosome organization (FDR of 3.6E−4), and the humoral immune response (FDR of 8.3E−6) (Fig. 2).

The distinguishing chemokine in the fatal-outcome group is interleukin-8 (IL-8) (CXCL-8), a proinflammatory chemokine involved in neutrophil degranulation and recruitment (24). In addition, IL-1RA, tumor necrosis factor alpha (TNF-α), CCL2 (monocyte chemoattractant protein 1 [MCP-1]), and transforming growth factor α (TGF-α) showed upregulation in the plasma of fatalities.

Proteins upregulated in the survival group are involved in blood coagulation, fibrin clot formation (FDR of 7.49E−7), and platelet degranulation (FDR of 3.09E−8) (Table S3). In addition, several apolipoproteins, APOE, APOL1, APOA4, APOC3, and APOC4, were elevated in the plasma of the survivors, resulting in the enrichment of proteins associated with lipoprotein particle assembly (FDR of 1.67E−5) (25). APOC4 (fold change = −3.27; *P* value = 0.001) was the most significantly upregulated protein in plasma from the survivor group (26).

10.1128/mbio.00567-22.8 TABLE S3 Protein list associated with EVD viral loads. Download Table S3, XLSX file, 0.01 MB. Copyright © 2022 Viodé et al. 2022 Viodé et al. https://creativecommons.org/licenses/by/4.0/ This content is distributed under the terms of the Creative Commons Attribution 4.0 International license.

Our proteomic analysis of the first sample collected at admission to the ETU revealed early and distinct host immune response signatures for both EVD outcome groups. We next expanded our analysis of the host response to characterize pathways associated with the viral burden in EVD patients across all time points.

### Viral load differentiates between EVD survivors and fatalities.

The initial viral load in an EBV-infected patient influences the disease outcome (27). We stratified the 38 plasma samples according to the most recent corresponding viral load measurements (within 1 day) (Fig. 1B) as measured by PCR and recorded as cycle threshold (*C_T_*) values. The *C_T_* value-based stratification was as follows: low *C_T_* (*C_T_* ≤ 25)/high viral load, medium *C_T_* (25 < *C_T_* < 50)/medium viral load, and high *C_T_* (*C_T_* = 50)/very low or undetectable viral load. On average, viral loads increased in fatalities and decreased in survivors over the course of the disease; however, this correlation was not absolute (Fig. 1C). We performed 1-way analysis of variance (ANOVA) to identify those proteins with differential expression according to the three viral load categories. After Benjamini-Hochberg multiple-testing correction, we identified 84 proteins of significant differential abundances (Table S3), which were further analyzed using an unsupervised hierarchical clustering approach.

**FIG 3:**
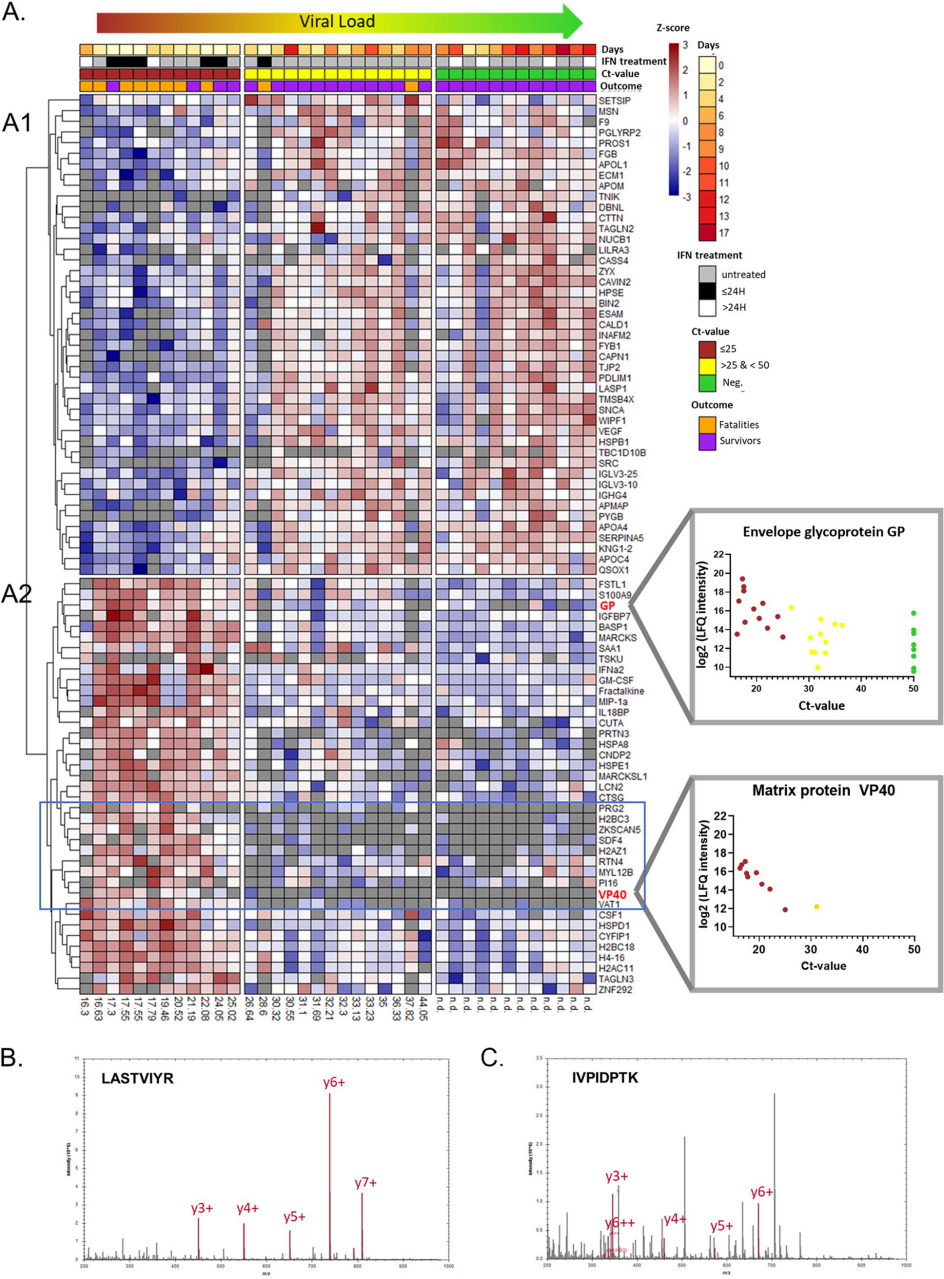
Viral load determines the proteome for EVD survivors and fatalities. (A) Hierarchical clustering of the 84 proteins exhibiting differential abundances among the three *C_T_* value groups, low *C_T_* value (≤25) (high viral load) (red), medium *C_T_* value (>25) (yellow), and high *C_T_* value (*C_T_* value = 50, i.e., viral load not detected) (green), based on 1-way ANOVA. Each row represents a protein, and each cell represents the protein abundance (Z-scored). Samples were ordered based on all collected *C_T_* values (ascending order). Two clusters of opposite trajectories were identified (A1 and A2). EBV proteins are highlighted in bold red. Correlations between the abundances of the EBV envelope glycoprotein (GP) and matrix protein VP40 [log_2_(LFQ intensity)] and the *C_T_* values are plotted on the right of the heatmap. n.d., not determined. (B) MS/MS spectrum of LASTVIYR 2+ (*m/z* 461.7715) for the EBV envelope GP. (C) MS/MS spectrum of IVPIDPTK 2+ (*m/z* 441.7684) for the EBV matrix protein VP40.

The heatmaps in Fig. 3A depict proteins whose expression levels are changing along with the *C_T_* value. We observed two major clusters: Fig. 3A1 describes 45 proteins whose expression increased as the viral load decreased, and Fig. 3A2 reveals 39 proteins whose expression levels increased with increasing viral loads. This analysis in Fig. 3A1 revealed several biological pathways associated with recovery from EVD (2, 28), i.e., upregulation of proteins linked to platelet degranulation (FDR of 1.1E−7), plasma lipoprotein particle assembly (FDR of 2.1E−3), regulation of body fluid levels (FDR of 7.1E−4), cell adhesion (FDR of 2.9E−4), and immune responses (FDR of 1.2E−3). Nine proteins are involved in the regulation of body fluid levels, such as kininogen 1 (KNG1), coagulation factor IX (F9), and fibrinogen beta chain (FGB). The cluster in Fig. 3A1 was enriched for actin cytoskeleton proteins (FDR of 5.6E−8) with the zyxin (ZYX), drebrin-like (DBNL), and bridging integrator 2 (BIN2) proteins.

Figure 3A2 reveals the above-mentioned 39 distinct proteins whose expression levels decreased as the viral load diminished; i.e., these proteins are associated with a more acute infection state. Biological pathways involved in leukocyte activation (FDR of 1.1E−5), nucleosome function (histones) (FDR of 8.1E−4), cytokine-mediated signaling (FDR of 8.0E−4), and inflammatory responses (FDR of 1.8E−2) were identified. In particular, the abundances of proteins associated with neutrophil extracellular traps (NETs) decreased with reductions in viral loads: cathepsin G (CTSG), myeloblastin (PRTN3), neutrophil gelatinase-associated lipocalin (LCN2), protein S100-A9 (S100A9), and core histones H2B, H2A, and H4 (29). Interestingly, a cluster of cytokines was also revealed, which included IFN-α2, granulocyte-macrophage colony-stimulating factor (GM-CSF), fractalkine (CX3CL1), chemokine (C-C motif) ligand 3 (CCL3/MIP1a), and IL-18 binding protein (IL-18BP).

Notably, our HTP-compatible biochemical depletion protocol enabled the detection of EBV-derived viral proteins in the plasma of EVD patients, namely, the envelope glycoprotein (GP), matrix protein VP40, and nucleoprotein (NP) (Fig. 3B and C; Fig. S2 to S4). NP was detected in only 6 samples, 5 of which had the highest viral loads. The expression levels of GP and VP40 decreased with decreasing viral loads. The envelope GP was detected even in plasma samples considered PCR negative for EBV.

10.1128/mbio.00567-22.3 FIG S2 EBV nucleoprotein (UniProt accession number O72142). (A) Run sequence coverage of 2% for the nucleoprotein (UniProt accession number O72142). Identified peptides are underlined with the corresponding *Q* values. (B) Mass spectrum of the precursor TPTVAPPAPVYR 2+ (amino acids 610 to 612). (C) Mass spectrum of the fragment ions obtained by DIA (high-energy collisional dissociation [HCD]). The corresponding fragment ions are highlighted in red and blue. Download FIG S2, TIF file, 1.6 MB. Copyright © 2022 Viodé et al. 2022 Viodé et al. https://creativecommons.org/licenses/by/4.0/ This content is distributed under the terms of the Creative Commons Attribution 4.0 International license.

Finally, we identified a heatmap cluster of proteins (indicated by the blue box in Fig. 3), whose expression levels mirror the VP40 expression pattern, suggesting that they may be directly interacting with this viral protein or even the viral particle. This cluster consists of 2 histones (H2BC3 and H2AZ1), zinc finger protein with KRAB and SCAN domains 5 (ZKSCAN5), and protein enriched in basophil (bone marrow proteoglycan [PRG2] and synaptic vesicle membrane protein VAT-1 homolog [VAT1]).

### EVD progression protein signatures in plasma.

**FIG 4:**
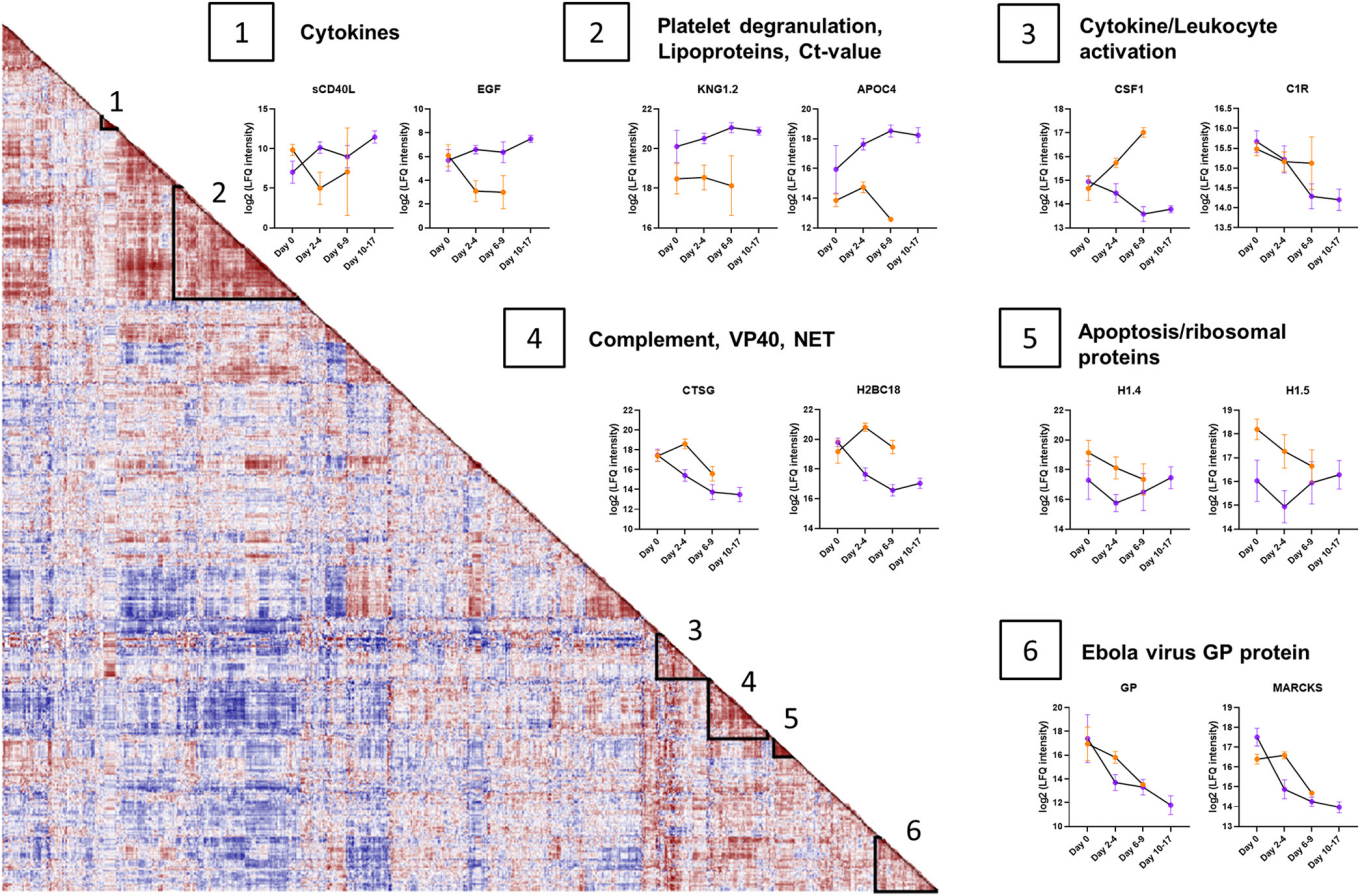
EVD progression signatures in the plasma proteome. Shown is a protein-protein correlation map over the course of the disease. Each cell represents the Pearson correlation coefficient (red, high; blue, low). Proteins were submitted to hierarchical clustering where six groups of proteins were identified. Longitudinal trajectories of the proteins of interest were plotted over the course of the disease (day 0, days 2 to 4, days 6 to 9, and days 10 to 17) for survivors (purple) and fatalities (orange). Group 1 comprises proteins with similar levels on day 0 that subsequently increase in survivors and decrease in fatalities. Protein levels are represented as log_2_(Label-free quantification (LFQ) intensity) values. In group 2, expression levels have distinguishing trends between survivors and fatalities, with a constant upregulation in survivors. Group 3 proteins increase in fatalities and decrease in survivors. A similar trend is observed among group 4 proteins, where NET-related protein levels are decreasing in survivors while the complement protein C5 is steadily increasing in survivors. Group 5 comprises histones that are upregulated in fatalities and decrease over time. Group 6 proteins are characterized by decreasing expression levels over time for both survivors and fatalities and include the EBV protein GP (*n* = 12) (survivors are in purple; fatalities are in orange).

To better interrogate the pathophysiology of EVD and concurrent plasma proteome trajectories, all proteins were analyzed using Pearson correlation prior to hierarchical clustering (Fig. 4). Each cell represents a Pearson correlation coefficient for the trajectories of two proteins. This analysis allowed us to cast a wider net by omitting multiple-testing-correction statistical significance, which assumes independence. However, assuming independence is suboptimal at best when it comes to identifying coregulated proteins. We focused our analysis on six groups of correlated proteins, for which we investigated the temporal changes in fatalities (orange) and survivors (purple) by plotting the trajectories of representative proteins.

Groups 1 and 2 exhibit a divergent trajectory depending on the outcome, i.e., survival versus death. Protein group 1 is enriched for cytokines: soluble CD40 ligand (sCD40L) and epidermal growth factor (EGF). Despite comparable plasma levels immediately after admission to the ETU, their expression levels diverged, with reductions over time in the fatalities and increases in the survivors. Similar divergent trends were observed for the proteins in group 2, specifically proteins involved in platelet degranulation (KNG1) and apolipoproteins such as APOC4. However, KNG1 already showed an almost 4-fold difference in abundance at admission to the ETU, indicating its utility as a potential early prognostic marker.

In contrast to protein groups 1 and 2, the plasma abundances of the proteins in group 3 (macrophage colony-stimulating factor 1 [M-CSF1] and complement protein 1R [C1R]) were elevated in the fatalities yet diminished in the survivors. Protein group 3 is characterized by leukocyte activation (FDR of 2.3E−8) and inflammatory response (FDR of 6.3E−5) pathways. Protein group 4 revealed a trend similar to that of protein group 3: we observed a constant decrease for survivors and an increase followed by a decrease for fatalities for the last time point collected. This group included EBV protein VP40 and proteins associated with NETs, such as CTSG, PRTN3, and core histone H2.

The expression levels of proteins in group 5 were initially different for the two outcome groups but then converged during the course of EVD, regardless of the outcome. KEGG pathway analysis identified enrichment for both ribosomal (FDR of 5.4E−5) and apoptotic (FDR of 8.4E−7) pathways, revealing proteins such as linker histones (H1-4 and H1-5), high-mobility-group proteins B1 and B2, and vimentin (VIM). The expression levels of proteins in group 6 decrease over time, regardless of survival or death outcomes, e.g., EBV protein GP and myristoylated alanine-rich C-kinase substrate (MARCKS).

### IFN-β-1a treatment in EVD affects the plasma proteome.

**FIG 5:**
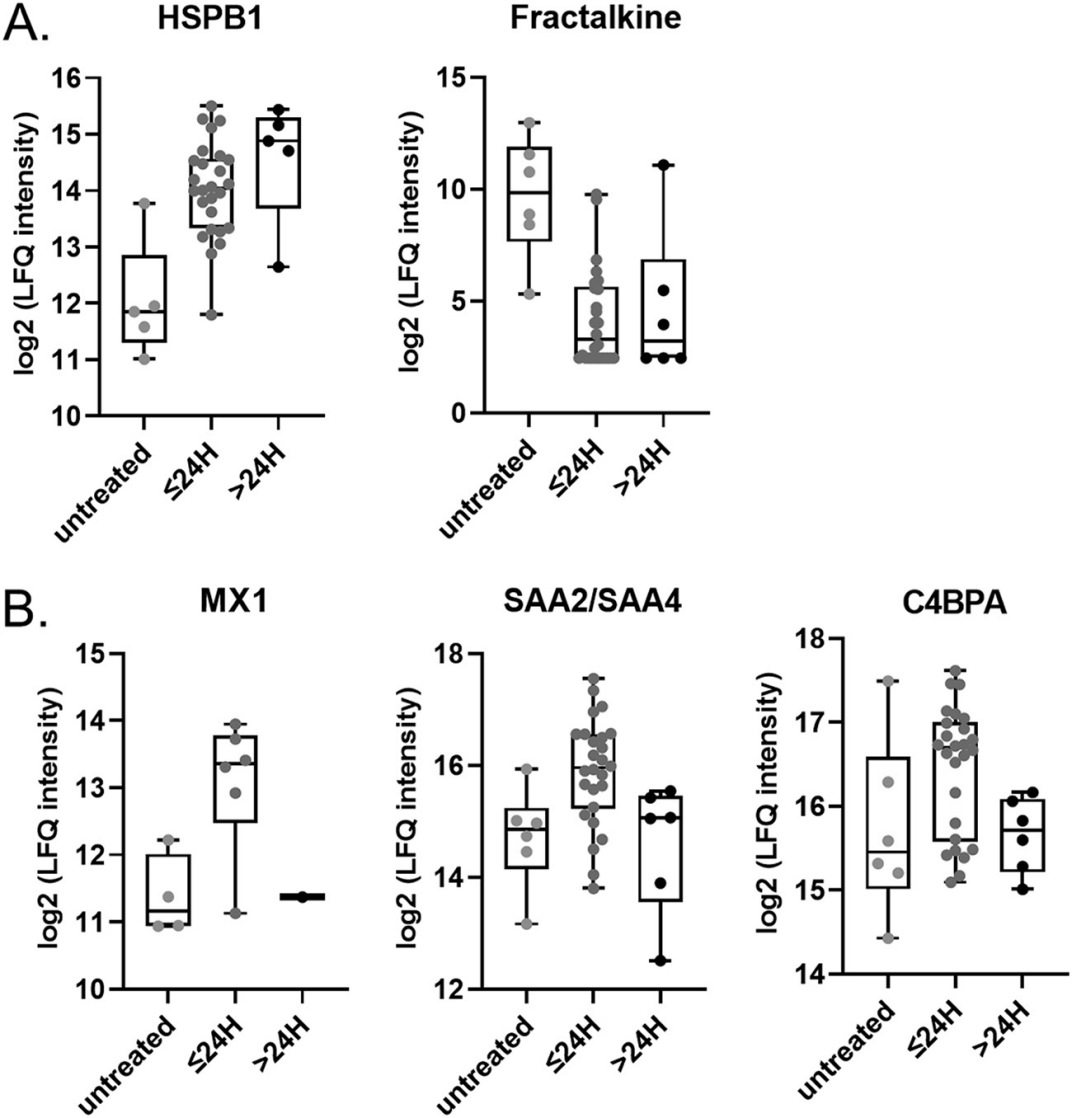
IFN-regulated proteins. Shown are log_2_ expression levels for the indicated proteins in plasma from untreated patients and plasma collected and analyzed at ≤24 h and ≥24 h relative to IFN treatment. One-way ANOVA was performed. (A) HSPB1 (*q* value = 0.045) and fractalkine (*q* value = 0.045); (B) MX1 (*q* value = 0.27), SAA2/SAA4 (*q* value = 0.21), and C4BPA (*q* value = 0.33).

To examine the effects of IFN-β-1a treatment on the plasma proteome, we subdivided the cohort into 3 groups, untreated (pretreatment group) (*n* = 6), ≤24 h after treatment (within 0 to 24 h after IFN-β-1a treatment) (*n* = 26), and >24 h after treatment (more than 24 h after IFN-β-1a treatment) (*n* = 6), and performed 1-way ANOVA. Our analysis identified two proteins whose expression levels changed significantly in response to treatment, namely, heat shock protein β1 (HSPB1) and fractalkine (CX3CR1). Whereas HSPB1 levels increased after IFN treatment, fractalkine levels decreased (Fig. 5A). We observed a similar pattern in protein expression levels for the IFN-inducible Mx protein (MX1), the serum amyloid proteins SAA2 and SAA4, and the complement inhibitor protein C4BPA, namely, an early increase in expression followed by a decline (Fig. 5B). Interestingly, MX1 protein expression clustered alongside EBV protein VP40, NET, and complement proteins in cluster 4 (Fig. 4), from which we infer that IFN-regulated MX1 expression levels may correlate with changes/course of EVD.

### Pathogen and host biomarkers are predictive of EVD outcome.

**FIG 6:**
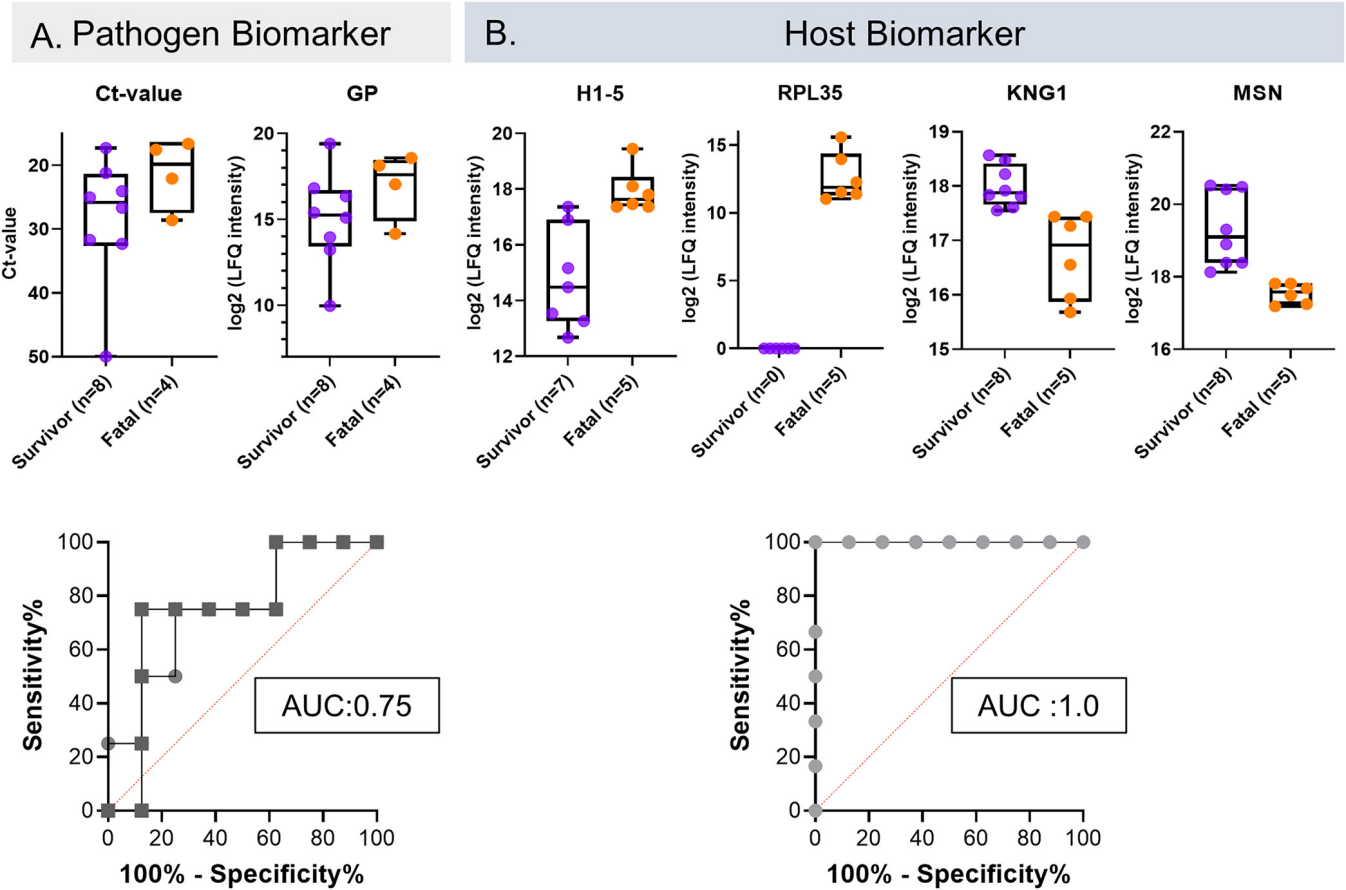
Pathogen and host protein biomarkers that are predictive of outcomes at admission. (A) Box plots of *C_T_* values (*P* value = 0.17) and EBV GP (*P* value = 0.17) expression levels segregated according to patient outcomes (survival or fatality). Both pathogen biomarkers have similar profiles, with an area under the receiver operating characteristic curve (AUROC) value of 0.75 (circles, *C_T_* values; squares, EBV GP). (B) Box plots of H1-5, RPL35, MSN, and KNG1 protein expression levels at admission, segregated according to patient outcomes. Each host biomarker protein identified has an AUROC value of 1.00, with a value of 1.00 being perfect accuracy (*P* value = 0.0027 for H1.5, *P* value = 0.0019 for MSN, and *P* value = 0.0019 for KNG1) (*n* = 12) (survivors are in purple; fatalities are in orange).

While the initial viral loads and levels of circulating viral proteins are reasonable predictors of the severity of EVD (Fig. 6A), host factors are also critical determinants of disease outcomes. Our data showed that the *C_T_* values and expression levels of EBV GP were generally predictive of outcomes in our EVD cohort, with an area under the receiver operating characteristic curve (AUROC) value of 0.75 (a value of 1.0 indicates perfect accuracy) (Fig. 6A), in agreement with previous studies (27, 30).

We identified four host immune response proteins (Fig. 6B) whose individual expression levels could perfectly predict EVD outcomes in the context of survival or death (i.e., AUROC value = 1.0), namely, histone H1-5 (*P* = 2.7E−3) (upregulated in fatalities), ribosomal protein RPL35 (*P* = 7.8E−4) (upregulated in fatalities), moesin (MSN) (*P* = 1.9E−3), and KNG1 (*P* = 1.9E−3), the latter two of which are upregulated in survivors. The statistical analysis approach used disregards missing values, i.e., instances in which proteins were not detected. Accordingly, we also performed a Fisher exact test to compare the numbers of missing values for each protein between fatalities and survivors. The results of this analysis are shown in Table S4. Seven out of nine proteins that we identified significantly more frequently in the plasma from fatalities than in the plasma from survivors are intracellular proteins such as ribosomal proteins and high-mobility proteins. These proteins were present in at least 4 of 5 fatality samples and were present in only 1 or 2 of the 8 survivor samples. Notably, RPL35 was detected exclusively in the plasma from fatalities, i.e., in 100% of the fatalities, but never in the plasma from survivors. Four of the five proteins identified to be more frequently observed in the plasma of the survivor group are associated with metabolic pathways, including CKM, ENPP3, ENO3, and PYGB.

10.1128/mbio.00567-22.9 TABLE S4 Markers found primarily in the fatality or survivor group. Shown are proteins identified in either the fatality or survivor outcome group at the initial time point based on a Fisher exact test. These proteins are expressed at a higher percentage in one of the outcome groups but not the other. Proteins associated with fatalities are mainly intracellular non-membrane-bounded organelle proteins. Proteins associated with survival are linked to metabolic pathways. Proteins identified in fatalities are the 60S ribosomal protein L35 (RPL35), high-mobility-group protein HMG-I/HMG-Y (HMGA1), apoptosis-associated speck-like protein containing a CARD (PYCARD), 60S ribosomal protein L7a (RPL7A), nucleophosmin (NPM1), nonhistone chromosomal protein HMG-17 (HMGN2), interleukin enhancer binding factor 3 (ILF3), and TBC1 domain family member 10B (TBC1D10B). Proteins identified in survivors are protein FAM228B, ectonucleotide pyrophosphatase/phosphodiesterase family member 3 (ENPP3), creatine kinase M type (CKM), beta-enolase (ENO3), and glycogen phosphorylase, brain form (PYGB). Missing values are calculated as the number of experimental observations divided by the number of theoretical observations, i.e., the number of patients in a group. Download Table S4, XLSX file, 0.01 MB. Copyright © 2022 Viodé et al. 2022 Viodé et al. https://creativecommons.org/licenses/by/4.0/ This content is distributed under the terms of the Creative Commons Attribution 4.0 International license.

## DISCUSSION

This study interrogated the plasma of EVD cases in Guinea during the 2013–2016 Ebola outbreak. We analyzed 38 plasma samples from 12 patients, of whom 8 survived and 4 died of EVD. Death occurred within a week of admission, while survivors were monitored for up to 3 weeks. We performed a plasma proteomic analysis on this unique EVD cohort, which included classical plasma proteins, tissue leakage-derived plasma proteins, and cytokines and chemokines. We developed a cost-effective HTP LC-MS-based plasma proteomics pipeline based on perchloric acid protein precipitation, which robustly removes the abundant plasma proteins and allows the detection of less abundant plasma proteins. While several proteomic studies have employed perchloric acid precipitation as a semiselective depletion/enrichment strategy (31–33) on small numbers of samples, we have advanced this protocol into a robust HTP-compatible large-scale plasma proteomic method. The main advantages of this depletion method include (i) the extreme precipitation conditions, which make the protocol impervious to variations in the starting conditions of the samples; (ii) the low cost per sample; and (iii) its compatibility with 96-well plates and liquid-handling robots. This method has proven its robustness for thousands of plasma samples in the Immunophenotyping Assessment in a COVID-19 Cohort (IMPACC) study (18). In the context of an acute viral infection, the perchloric acid method has the additional advantage of enabling the detection of the pathogen, i.e., EBV-derived proteins and the enrichment of histones that are important for the host response to viral infection.

We quantified over 1,000 proteins across our three protein platforms, which allowed us to characterize the differences in the early stages of the host response and the outcome-specific trajectories, clearly differentiating between fatalities and survivors (upon admission) (Fig. 2). In this analysis, we observed distinct plasma protein patterns when comparing patients who succumbed to EVD to survivors, revealing early protein signatures and immune pathways that differentiated survivors and fatalities at the time of admission and during the course of the infection.

Furthermore, we interrogated protein profiles for correlations with viral loads and over the course of the disease (Fig. 3 and 4). To this end, we performed (i) an ANOVA-based analysis stratifying the samples into groups based on the *C_T_* value to examine the association between the viral load and the host proteome (Fig. 3) and (ii) a protein-protein correlation map based on Pearson correlation prior to hierarchical clustering of the proteins and studied the temporal changes of groups of coregulated proteins in both fatalities and survivors (Fig. 4). From the results of these analyses, we derived prognostic protein biomarkers that may differentiate between fatalities and survivors with better performance than the viral load alone. Finally, we assessed the effect of IFN-β treatment on the plasma proteome.

Upon admission, the plasma proteome of fatalities is characterized by an upregulation of proteins involved in the immune response (complement proteins and cytokines), nucleosome organization (histones), and the regulation of gene expression (histones and ribosomal proteins) (Fig. 2). Complement exerts a key role in the response to viral infection, modulating the proinflammatory response (34). Similarly, CXCL-8, TNF-α, CCL2, and TGF-α are produced by several immune cell subtypes, including macrophages, which are known to be activated in EVD (10) and secrete proinflammatory cytokines (35–37). CXCL-8 and TNF-α are directly involved in the recruitment of additional macrophages to the infection site, which results in the breaking of the endothelial barrier (37). Moreover, CXCL-8, TNF-α, and CCL2 are markers of sepsis (38, 39).

Regardless of the outcome, a high viral load is characterized by an increase in inflammatory markers (40), specifically cytokines, chemokines, and complement cascade components. Cytokines and chemokines exert pleiotropic effects on the innate immune response, inducing protective local and systemic responses, both pro- and anti-inflammatory responses, and regulating the adaptive immune response (40–43). The expression levels of CSF1, GM-CSF, CCL3, CX3CL1, and IL-18BP were elevated with high viral loads (Fig. 3). Notably, CSF1 levels increased over time in fatalities while decreasing in survivors (Fig. 4, panel 3). Pathological cytokine and chemokine release leads to endothelial cell toxicity and, ultimately, vascular cell leakage (44); we found an upregulation of proteins involved in blood coagulation, fibrin clot formation, as well as the actin cytoskeleton in survivors. The complement system, involved in the response to viral infection, triggers a protease cascade, regulates an inflammatory response (34), and shapes the humoral immune response. In this study, we identified C2 and C5 levels to be upregulated in fatalities.

Histones were among the most prominent proteins upregulated early in our cohort of fatalities with low *C_T_* values (high viral loads) (Fig. 2 and 3). Histones arise from cell leakage upon tissue damage but may also be a result of regulated release by NETs (45). Irrespective of their origin, circulating histones have been associated with microbicidal activity, limiting the spread of infection or injury (46). Concurrently, uncontrolled levels of histones can have toxic effects (46): extracellular histones are elevated in sepsis (47) and are associated with tissue and multiple-organ injury (48) and/or death (49, 50). These findings are consistent with our observation that markedly elevated levels of histones are associated with fatalities in EVD (10), supporting the potential for developing histone-targeting therapeutics (47). Our data suggest that the histones detected likely arose from NETs, as we detected other dysregulated markers of NETs, including myeloperoxidase (MPO), cathepsin G (CTSG) (Fig. 4, panel 4), and proteinase 3 (PRTN3). These proteins suggest the presence of NETs in the blood of EVD patients, specifically in those with a poor prognosis (Fig. 3), further suggesting that NET dysregulation may be harmful, leading to organ injury (46, 48). In short, similar to extracellular histones, NETosis must be tightly regulated to prevent NET dysregulation associated with excessive tissue damage during acute or chronic infections. Our findings are consistent with those of Eisfeld et al. (10), who suggested that NETs play a key role in the pathogenesis of EVD. In survivors, levels of CTSG and histone H2B type 2-F (H2BC18) decline rapidly compared to the marked increase in fatalities (Fig. 4, panel 4). Our data suggest that EVD induces the production of NETs, which are beneficial for viral clearance, but their overexpression leads to tissue damage by circulating histones (51, 52).

In contrast to the signature protein profiles identified in the fatalities, proteins upregulated in survivors upon ETU admission are associated with low viral loads (high *C_T_* values), platelet degranulation, blood coagulation, fibrin clot formation, and plasma lipoprotein particle assembly (Fig. 2 to 4), i.e., the disease resolution pathway. One of the hallmarks of EVD is vascular leakage and endothelial cell disruption (2), which is limited/prevented by the upregulation of blood coagulation factors in EVD survivors, as previously described for recovery after vascular injury (53, 54) and observed in our cohort. Platelet degranulation proteins (55) and apolipoproteins, which we observed to be upregulated in survivors, i.e., good prognosis (Fig. 4), have been associated with antiviral activity (56, 57). Notably, apolipoproteins are critical effectors in the immunomodulation of EVD pathogenesis (58) and other infectious diseases (including COVID-19 [59]), where decreased levels of apolipoproteins are associated with sepsis (60).

Interestingly, the composite protein signature of EVD survivors included proteins associated with actin cytoskeleton formation. The intact actin cytoskeleton, critical for normal cellular function, is altered during sepsis (61). The positive correlation between *C_T_* values and host proteins involved in actin cytoskeleton formation and platelet degranulation (Fig. 3) strongly suggests that the control/clearance of the virus is associated with the functional upregulation of wound repair (2, 28), while the depletion of actin-associated proteins in the EVD fatality patients may reflect hemorrhagic events and damage to the integrity of the endothelium (2).

Our HTP depletion protocol allowed us to detect the EBV proteins NP, GP, and VP40. GP and VP40 protein levels measured by MS correlated with *C_T_* values (Fig. 3). Interestingly, GP, the only protein expressed on the virus surface, was detected even in samples with *C_T_* values of 50, i.e., samples considered PCR negative for EBV. The detection of GP in PCR-negative samples may reflect late-stage EVD (62), slow protein turnover (63), or even viral persistence albeit undetectable by PCR, as demonstrated previously in ocular fluid (64) and semen (65–67). In contrast, VP40 was detected only in samples with the highest viral loads and was undetectable in samples with a *C_T_* of >25 (with one exception). VP40 showed an excellent negative correlation with the *C_T_* value (*R* = −0.91), indicating that VP40 is likely a more accurate reflection of the actual blood-borne viral load than GP, which seems to be more stable and lingers for a longer time. Although frequently an indicator of potential outcomes (68), the viral load upon the onset of EVD does not always predict the severity of the disease. Indeed, EVD patients 6 and 10 in our cohort both succumbed to the disease despite their low viral loads: patient 6 showed a progressively decreased viral load (Fig. 1C), and patient 10 had a low viral load at admission.

A number of studies have attempted to identify early biomarkers predictive of outcome, using mRNA (30) or a multi-OMICs analysis of EVD (10). mRNA studies revealed a panel of 10 genes as predictors of survival versus fatality, with an accuracy of 92%. The multi-OMICs study identified 11 distinct biomarkers (10). However, given the resource limitations in regions at risk for EBV outbreaks, the likelihood of using these biomarkers/technologies for disease prognosis is low. In contrast, we propose a biomarker panel comprising four host proteins that are distinguishable upon the onset of symptoms, which coincided with ETU admission, with individual AUROC values of 1.0. The expression levels of these proteins were predictive of EVD outcomes, including (i) H1-5 protein, a linker histone associated with organ injury (48); (ii) MSN, which regulates the structure and function of the cell cortex (69); and (iii) KNG1, involved in blood coagulation and platelet degranulation (70). Additionally, we identified the ribosomal protein RPL35 as a highly promising biomarker, as it was always observed in the samples of the fatalities but was never observed in the samples of the survivors. Ribosomal proteins are involved in the innate immune response and participate in the regulation of the inflammatory response (71). Interestingly, the plasma samples of two patients, patients 6 and 10, who would have been misclassified if one had focused on their low viral loads/*C_T_* values alone, presented with the plasma protein signature associated with a fatal outcome. In short, the functional link between the components of our proposed biomarker panel and severe viral infections and organ injury is well established, thereby meeting the strict requirements for biomarker candidacy. The biomarkers identified, such as histone H1-5, involved in NETosis, are likely to be reflective of severe viral infection and associated organ injury and cell death. By focusing on protein biomarkers, their measurement can be easily evaluated, using dipsticks, lateral flow immunoassays, or paper-based diagnostics (72). All of these assay formats are rapid, reliable, and field compatible.

Limitations of this study were the small cohort size and the one-arm clinical study design. For compassionate reasons, all patients were treated with IFN-β-1a, resulting in no access to matched, untreated infected plasma samples. This limitation restricted our analyses of (i) the effects of IFN-β-1a on the plasma proteome and (ii) the impact of treatment on the two outcome groups. However, clinical study data (15) did identify benefits of IFN treatment, namely, earlier resolution of the disease and improved survival rates. Despite the limitations of a small cohort size, with limited samples, our findings reveal signature protein profiles in plasma from EVD patients that distinguish between fatalities and survivors. Notably, we identified 4 protein biomarkers that differentiate outcomes with better accuracy than the viral load at admission. Analytical validation of these host biomarkers, i.e., measuring the limit of detection (LOD)/limit of quantification (LOQ), could be performed using quantitative targeted LC-MS methods. These findings will need to be confirmed with an independent cohort.

## MATERIALS AND METHODS

### Cohort design and patient characterization.

The initial study population involved patients who were admitted to the Ebola Treatment Unit (ETU) in a rural area close to the town of Coyah in West Guinea, during the period of 26 March to 12 June 2015. This was a single-arm clinical study to evaluate the effectiveness of IFN-β-1a against EVD (12). Inclusion (eligibility) criteria for IFN-β-1a therapy were (i) symptom onset within 6 days, (ii) blood real-time reverse transcription-polymerase chain reaction (RT-PCR)-confirmed positivity for EBV, and (iii) patient/designee informed consent for the use of IFN-β-1a. Exclusion criteria included (i) symptom onset more than 6 days prior to admission, (ii) age of <17 or >70 years, and (iii) contraindication for the use of IFN-β-1a or any of the constituents of the drug product. See the supplemental material for details on ethics approval.

A total of 38 samples were collected from 12 PCR-confirmed EVD patients, 9 of whom received IFN-β-1a treatment (see Table S1 in the supplemental material). Peripheral blood plasma was collected, approximately every 2 to 4 days, during the course of the disease, prior to and after treatment, until discharge or death. All plasma samples were stored at −80°C. The samples were inactivated by gamma irradiation before shipment to the processing laboratories. Local and international IRB committees approved all protocols utilized. Approvals were obtained from the Guinean Ministry of Health (#0777/CNRE; Dr. Sakoba Keita) (February 29, 2015), the CNERS, Guinea (016/CNERS/15; Prof. Oumou Younoussa Sow) (February 16,2015) and the Ebola Research Commission, National Public Health Institute, Guinea (Dr. Lamine Koivogui) (December 12, 2014). Written informed consent was obtained from all patients who received IFN β-1a treatment. The trial registration is: ISRCTN 17414946. The Guinean Health Ministry registered the trial as #0777/CNRE on February 29, 2015. All patients had tested positive for EVD at the time of admission to the field ETU (Fig. 1A; see the supplemental material for further details).

### Sample preparation. (i) Classical plasma proteomics (Cl-PP).

Samples were processed using our in-house MStern blotting protocol (20, 73). In brief, 1 μL of plasma was mixed with 100 μL of urea buffer. Following the reduction and alkylation of cysteine residues, 10 to 15 μg of proteins was loaded into individual wells of a 96-well plate containing a polyvinylidene fluoride (PVDF) membrane (Millipore-Sigma). Protein digestion using trypsin was performed for 2 h at 37°C, and the tryptic peptides were eluted from the membrane using 40% acetonitrile (ACN)–0.1% formic acid (FA). Peptides were desalted using a 96-well Macrospin C_18_ plate (Targa; NestGroup), and the eluents were dried in a vacuum centrifuge and stored at −20°C until liquid chromatography-tandem mass spectrometry (LC-MS/MS) analysis.

### (ii) Tissue leakage plasma proteomics (TL-PP).

Twenty-five microliters of plasma was diluted with 475 μL of water, 5% of perchloric acid (25 μL) was added, and after vigorous agitation, the mixture was kept on ice for 15 min. Samples were then centrifuged for 15 min (4°C at 16,000 × *g*), and the supernatant was aspirated, mixed with 50 μL of 1% trifluoroacetic acid (TFA), loaded onto a solid phase extraction (SPE) Hydrophilic-Lipophilic-Balanced (HLB) 96-well μElution plate previously conditioned with 300 μL methanol, and mixed two times with 500 μL of 0.1% TFA for desalting and the removal of perchloric acid. The samples were then digested with 500 ng of trypsin overnight at 37°C and resuspended in 50 μL of 0.1% formic acid, and 4 μL was injected into the LC-MS instrument.

### DDA and DIA sample acquisition.

Samples were injected on the same LC-MS system (Eksigent 400 series ultraperformance liquid chromatography [UPLC] system [Sciex] combined with Q Exactive [Thermo Scientific]) for data-dependent acquisition (DDA) and data-independent acquisition (DIA). Peptides were separated on a PicoChip column (Acquity BEH C_18_, 150 μm by 100 mm, 1.7 μm; New Objective) with a 45-min gradient and a total run time of 58 min. For the DIA experiment, 15 variable windows were used over the *m/z* range of 375 to 1,200. More details can be found in Text S1 in the supplemental material.

10.1128/mbio.00567-22.1 TEXT S1 Supplemental methods. Download Text S1, DOCX file, 0.02 MB. Copyright © 2022 Viodé et al. 2022 Viodé et al. https://creativecommons.org/licenses/by/4.0/ This content is distributed under the terms of the Creative Commons Attribution 4.0 International license.

### Cytokine/chemokine analysis.

Cytokine and chemokine levels were measured using a Milliplex human cytokine/chemokine magnetic bead premixed 41-plex kit (catalog number HCYTMAG-60K-PX41; Millipore) on the plasma collected from the EVD cohort, as previously described (74). More details can be found in Text S1.

### Statistical analyses.

The protein abundance matrices from Spectronaut v12 (Biognosys, Switzerland) obtained for both protocols were combined. For the proteins shared between Cl-PP and TL-PP, we used the quantifications from the platform with the lower number of missing values. Cytokine/chemokine data were then added to that matrix to obtain a unique protein/LFQ intensity matrix for all the platforms. Statistical analysis was performed using Perseus software and R Studio. We applied the parametric Student *t* test on the log_2_-transformed LFQ intensity of the proteins with a *P* value of 0.05 as a cutoff. Pathway analysis for the differentially expressed proteins was undertaken using the STRING interaction network.

### Data availability.

To ensure public availability, deidentified data described in this article, including plasma protein and cytokine/chemokine concentrations, were archived at the PRIDE ProteomeXchange under the following accession numbers: PXD030260 (http://www.ebi.ac.uk/pride/archive/projects/PXD030260).

10.1128/mbio.00567-22.4 FIG S3 EBV envelope glycoprotein (UniProt accession number O11457). (A) Run sequence coverage of 15% for the envelope glycoprotein (UniProt accession number O11457). Identified peptides are underlined with the corresponding *Q* values. (B) Mass spectrum of the precursor LASTVIYR 2+ (amino acids 165 to 172). (C) Mass spectrum of the fragment ions obtained by DIA (HCD). The corresponding fragment ions are highlighted in red and blue. Download FIG S3, TIF file, 1.4 MB. Copyright © 2022 Viodé et al. 2022 Viodé et al. https://creativecommons.org/licenses/by/4.0/ This content is distributed under the terms of the Creative Commons Attribution 4.0 International license.

10.1128/mbio.00567-22.5 FIG S4 EBV matrix protein VP40 (UniProt accession number Q05128). (A) Run sequence coverage of 15% for VP40 (UniProt accession number Q05128). Identified peptides are underlined with the corresponding *Q* values. (B) Mass spectrum of the precursor IVPIDTK 2+ (amino acids 249 to 256). (C) Mass spectrum of the fragment ions obtained by DIA (HCD). The corresponding fragment ions are highlighted in red and blue. Download FIG S4, TIF file, 1.1 MB. Copyright © 2022 Viodé et al. 2022 Viodé et al. https://creativecommons.org/licenses/by/4.0/ This content is distributed under the terms of the Creative Commons Attribution 4.0 International license.
