# Supplementary figures and images for: Plasma Proteomic Analysis Distinguishes Severity Outcomes of Human Ebola Virus Disease
Source: mBio. 2022 Apr 21;13(3):e00567-22. doi: 10.1128/mbio.00567-22 (PMC9239184; doi:10.1128/mbio.00567-22)

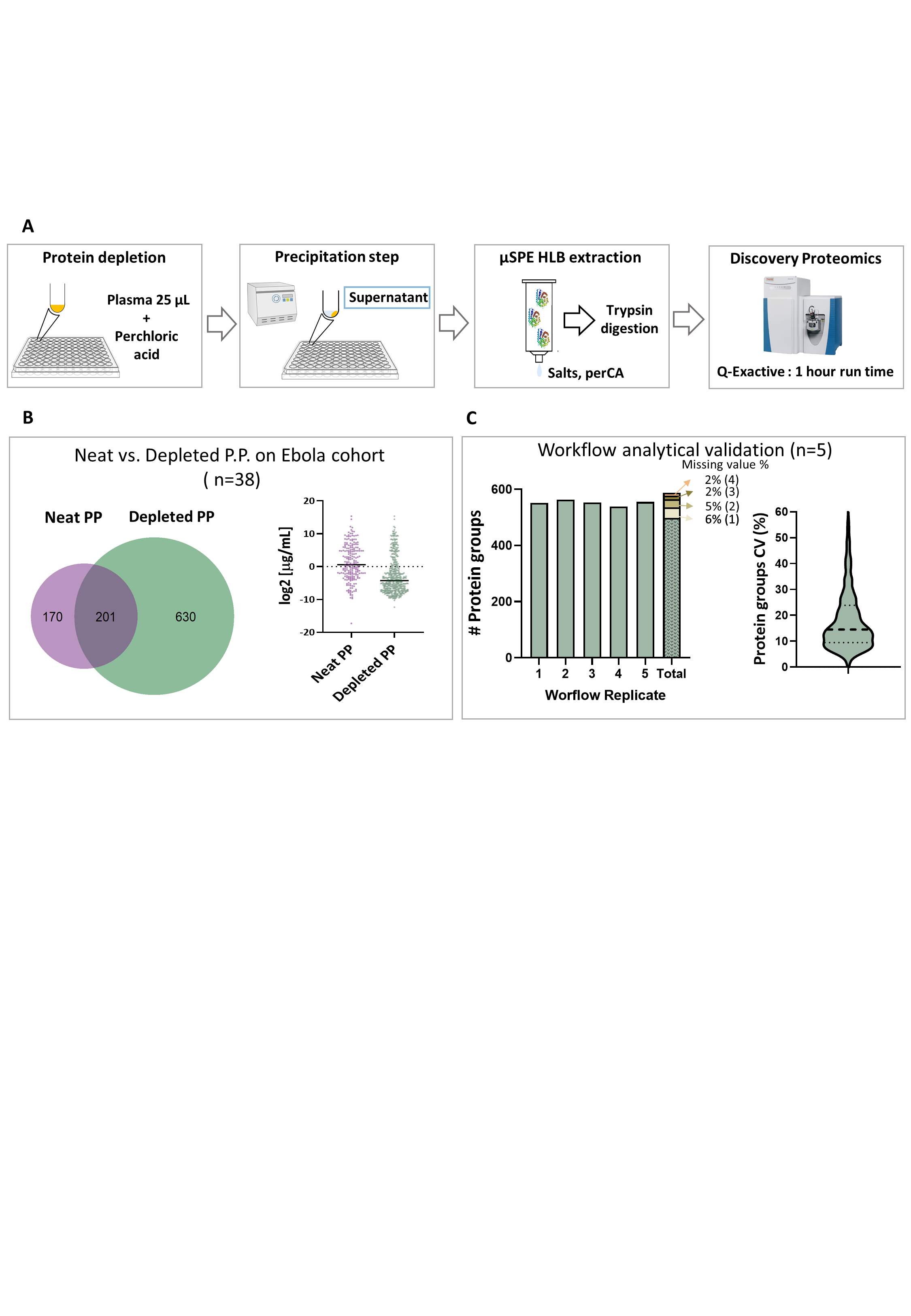

Supplement: FIG S1 [file mbio.00567-22-s0002.tif]

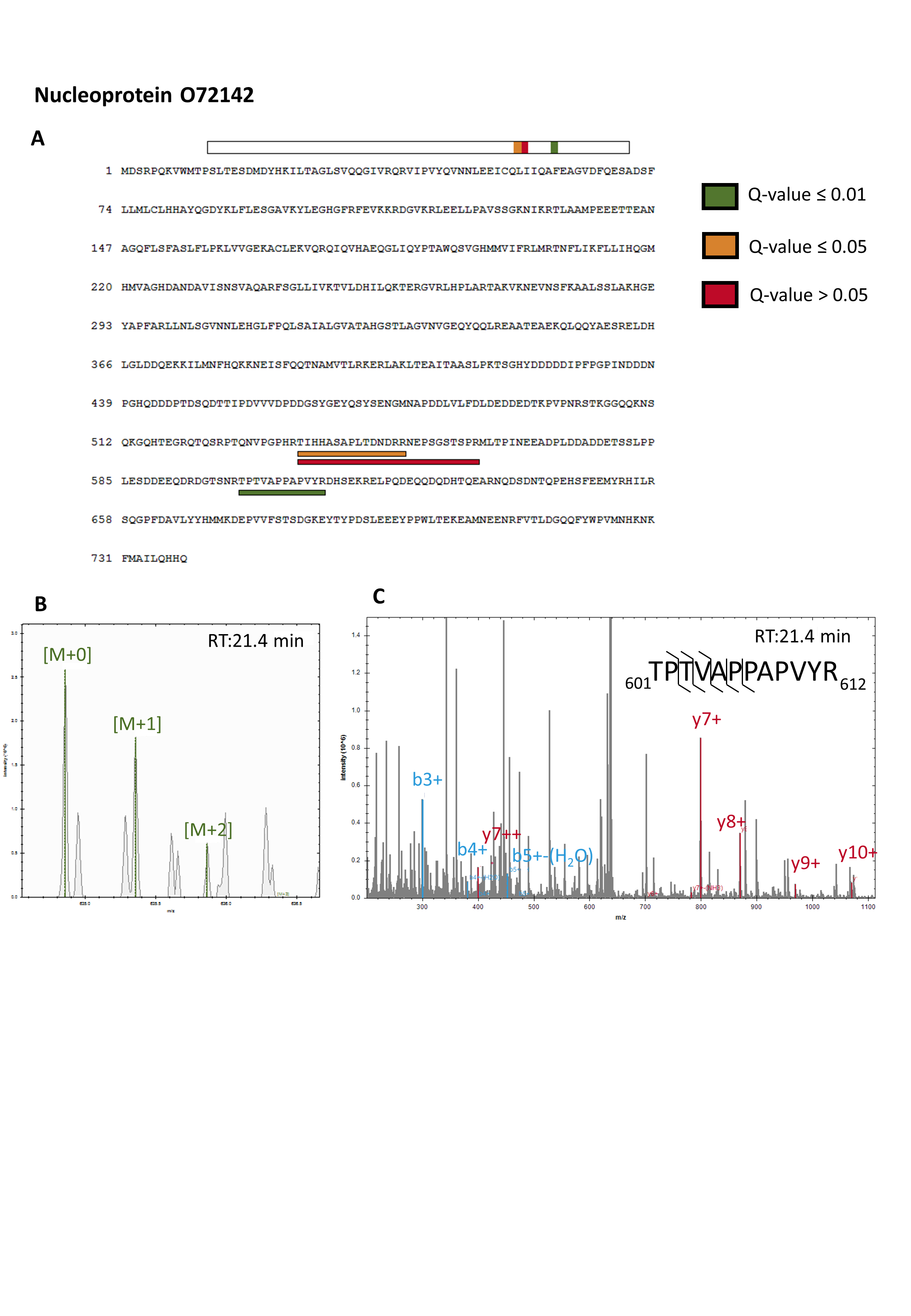

Supplement: FIG S2 [file mbio.00567-22-s0003.tif]

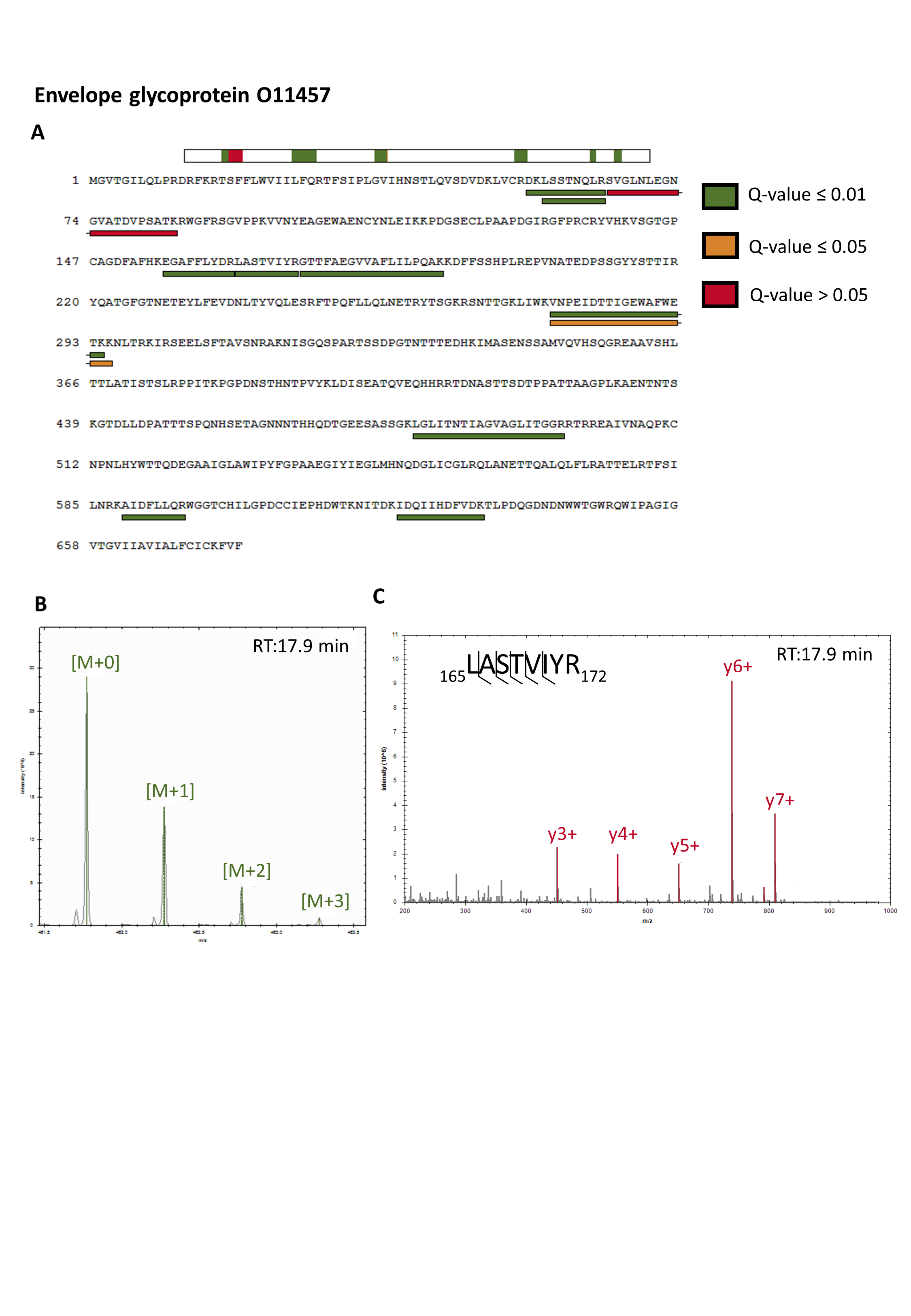

Supplement: FIG S3 [file mbio.00567-22-s0004.tif]

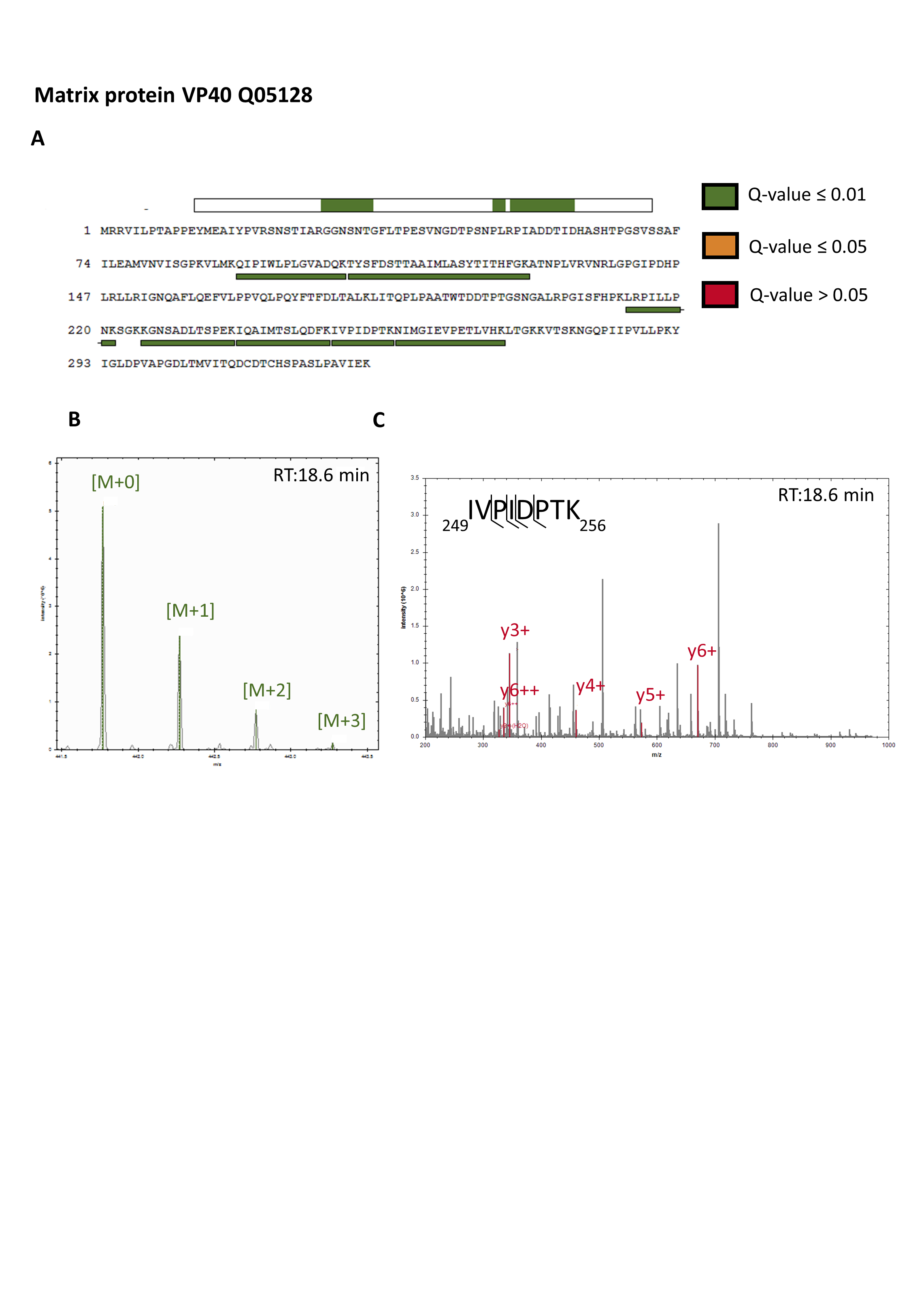

Supplement: FIG S4 [file mbio.00567-22-s0005.tif]
