# Supplementary material for: Plasma Proteomic Analysis Distinguishes Severity Outcomes of Human Ebola Virus Disease
Source: mBio. 2022 Apr 21;13(3):e00567-22. doi: 10.1128/mbio.00567-22 (PMC9239184; doi:10.1128/mbio.00567-22)
Supplement: TEXT S1 [file mbio.00567-22-s0001.docx]

### **Human Ebola virus disease: plasma proteomic analysis distinguishes severity outcomes**

Arthur Viodé, Kinga K Smolen, Benoit Fatou, Zainab Wurie, Patrick Van Zalm, Mandy Kader Konde, Balla Moussa Keita, Richard Amento Ablam, Eleanor N. Fish and Hanno Steen

## Supplementary Materials and Methods:

**Ethics statement:**

This study was conducted in accordance with Good Clinical Practice Guidelines and the Declaration of Helsinki. Approvals were obtained from the Guinean Ministry of Health (#0777/CNRE; Dr. Sakoba Keita) (February 29, 2015), the CNERS, Guinea (016/CNERS/15; Prof. Oumou Younoussa Sow) (February 16, 2015) and the Ebola Research Commission, National Public Health Institute, Guinea (Dr. Lamine Koivogui) (December 12, 2014). Written informed consent was obtained from all patients who received IFN β-1a treatment. ISRCTN 17414946. The Guinean Health Ministry registered the trial as #0777/CNRE on February 29, 2015, i.e., the registration of this trial was delayed.

**Cohort design and patient characterization:**

Ct value were measured unsing a semi-quantitative RT-PCR assay which target the L gene of filoviruses (RealStar Filovirus Screen RT-PCR kit 1.0, altona Diagnostics, GmbH).

All patients had tested positive for EVD at time of admission to the field ETU (**Figure 1A)**. Purple = survivors (n = 8), orange = fatalities (n = 4)). Of the 12 enrolled patients, 8 were treated with IFN β-1a following admission to the ETU. Two IFN treated patients died within 48h of admission, and two untreated patients who were alive at the first sampling, subsequently died.

Age and sex of the EVD patients studied are described (**Figure 1B)**. An equal ratio of male (n=6) to female (n=6) patients were enrolled. Of the 12 patients enrolled, 8 (66.6%) recovered from the infection and 4 (33.3%) succumbed (**Figure 1A**&**B**). The average age for those who survived the infection was 29.8 years (20-50) and for those who died was 30.4 years (18-50). The average Ct value at day 0 was 23.3 (17.3-30.6) for survivors, and 24.6 (17.6 - 28.6) for those who died. Neither age nor Ct values were statistically different between those that survived and those that died. **Figure 1C and 1D** indicate the Ct level at day 0, i.e., 1^st^ IFN dose **(Figure 1D)**, and the changes over the course of infection **(Figure 1C)**.

**Spectral library acquisition and DIA data analysis:**

The mass spectrometer parameters were set as follows: resolution 35000 @ m/z 200, AGC target 3e6, maximum IT 120 ms, ﬁxed ﬁrst mass m/z 200, NCE 27. The DIA scans preceded an MS1 Full scan with identical parameters yielding a total cycle time of 2.4s.

The DDA runs were used to build the spectral library and the DIA were analyzed using Spectronaut v12 (Biognosys). Standard settings were employed. For the perchloric acid protocol, no modifications of cysteines were selected, as no alkylation reagent was used in the protocol. We combined the DDA from previously published plasma/serum proteomics studies to use as the spectral NeatPP library for this study. Only the DDA runs of this study were used to build the DepletedPP library.

**Cytokine/chemokine analysis:**

The cytokine/chemokines kit measures the following analytes: sCD40L, EGF, FGF-2, Flt-3 ligand, Fractalkine, G-CSF, GM-CSF, GRO (CXCL1), IFN-α2, IFN-γ, IL-1α, IL-1β, IL-1ra, IL-2, IL-3, IL-4, IL-5, IL-6, IL-7, IL-8 (CXCL8), IL-9, IL-10, IL-12 (p40), IL-12 (p70), IL-13, IL-15, IL-17A, IP-10 (CXCL10), MCP-1 (CCL2), MCP-3 (CCL7), MDC (CCL22), MIP-1α (CCL3), MIP-1β (CCL4), PDGF-AB/BB, RANTES (CCL5), TGF-α, TNF-α, TNF-β, VEGF, Eotaxin (CCL11), and PDGF-AA. Plasma stored at -80°C was thawed and centrifuged for 10 min at 3000 ×g. The samples were assayed according to the manufacturer’s instructions. Samples were analyzed using a Flexmap 3D system with Luminex xPONENT software (Luminex Corp.; Austin, TX, USA). A 5-parameter logistic plot was used to calculate the standard curve and the sample concentrations. The lower and upper limits of detection were set as the lowest and highest concentration of the standard curve, respectively. Samples with values below the limit of detection were assigned a value of the limit of quantification. If readings were less than 50 beads concentration, values were discarded for any sample or analyte. Samples with all analytes below the lower limit of detection were excluded from analysis.
